# Opioid Overdose Prevention Toolkit Released

**Published:** 2013-10-04

The Substance Abuse Mental Health Services Administration has released the Opioid Overdose Prevention Toolkit. The toolkit is the first federal resource promoting safety and prevention information for persons at risk for overdose, such as how to recognize and respond appropriately to overdose, specific drug-use behaviors to avoid, and the role of naloxone in preventing fatal overdose. The toolkit equips communities and local governments with material to develop policies and practices to help prevent and respond appropriately to opioid-related overdose. Prescribers will find evidence-based guidance for safe prescribing practices, identifying patients at risk for overdose, engaging them in prevention and risk-reduction efforts, and accessing opioid-dependence treatment. The toolkit is available at http://store.samhsa.gov/product/opioid-overdose-prevention-toolkit/all-new-products/sma13-4742.

